# Epigenetic alterations in canine mammary cancer

**DOI:** 10.1590/1678-4685-GMB-2022-0131

**Published:** 2022-10-24

**Authors:** Bárbara do Nascimento Borges

**Affiliations:** 1 Universidade Federal do Pará, Instituto de Ciências Biológicas Laboratório de Biologia Molecular, Belém, PA, Brazil.

**Keywords:** Histone modifications, DNA methylation, ncRNA, biomarkers, comparative oncology

## Abstract

In dogs, mammary cancer is the most common tumor type, especially in unspayed females. As in humans, this type of cancer has spontaneous development and is influenced by several risk factors, such as age and hormonal exposure in addition to genetic and epigenetic factors. Epigenetic mechanisms are responsible for gene expression modulation without alterations in the DNA sequence and include but are not limited to DNA methylation, histone modifications, and noncoding RNAs. Epigenetic patterns are known to influence a variety of biological mechanisms, such as cellular differentiation and development, and dysregulations of those patterns may result in several diseases, such as cancer. In this respect, this review summarizes the main findings concerning epigenetic alterations in canine mammary cancer, their relationship with the carcinogenic process, and their use as diagnostic and prognostic markers.

## Introduction

As in humans, mammary cancer is the most frequent cancer diagnosed among female dogs, especially unspayed dogs (Markkanen, 2019; Mohammed et al., 2020). This could be partly explained by dogs being a companion animal, sharing similar environmental conditions with humans (Rybicka et al., 2015; Bulkowska et al., 2017; Jeong et al., 2019). In addition, both species share several mammary cancer risk factors and biological patterns, including aging, hormonal exposure, obesity in early life, treatment response (due to similar P450 cytochrome activity), and spontaneous development (Rybicka *et al.*, 2015; Bulkowska *et al.*, 2017; Markkanen, 2019; Xavier et al., 2019; Beetch et al., 2020).

**Figure 1 -:**
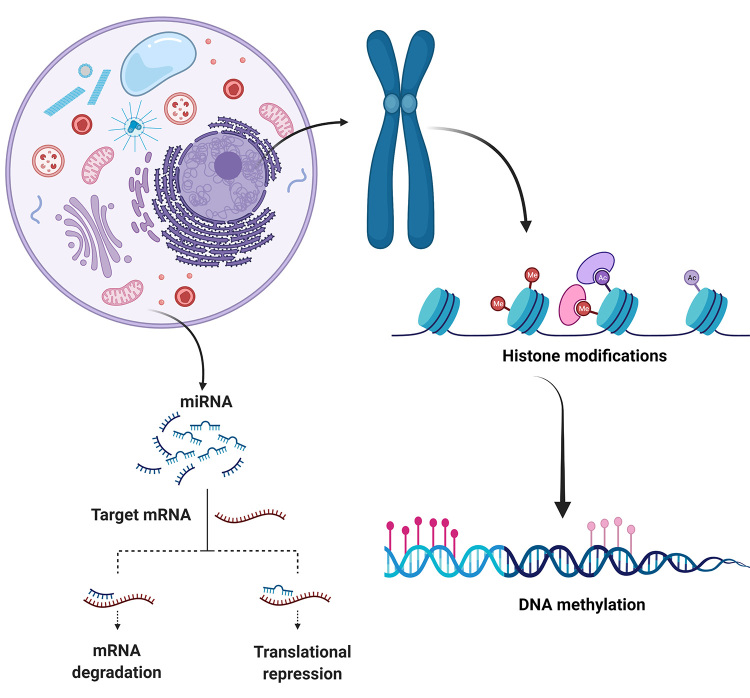
Main epigenetic mechanisms studied in canine mammary cancer: histone modifications, DNA methylation and miRNA expression. miRNAs may affect gene expression in two ways depending on their complementarity with the target messenger RNA (mRNA): perfect complementation results in mRNA cleavage, while partial complementation leads to translational repression.

Epigenetic mechanisms result in changes in gene expression without alterations in the DNA sequence (Inbar-Feigenberg et al., 2013; Zhou et al., 2021), influencing a variety of biological phenomena, such as cellular differentiation and development, metabolism, phenotypic variability, inheritance, evolution, behavior, and several diseases, such as cancer (Nicoglou and Merlin, 2017; Zhou *et al.*, 2021). The regulatory mechanisms of epigenetic modifications include DNA methylation, RNA interference, histone modifications (Figure 1) (Zhou *et al.*, 2021), DNA‒protein interactions, chromatin accessibility and tridimensional structure (Casado-Pelaez et al., 2022).

Although mutations in many genes, such as in *BRCA1*, *BRCA2*, and *TP53* (Markkanen, 2019; Beetch et al., 2020), as well as changes in several mammary cancer-related pathways, such as *KRAS*, *PTEN*, and *MAPK* (Bulkowska et al., 2017), have already been described for both species, the genetic influence in the genesis and development of this tumor and information about epigenetic alterations in canine mammary cancer are still scarcely known. The aim of this review is to summarize the main findings concerning epigenetic alterations in canine mammary cancer, their relationships with carcinogenesis and their use as diagnostic and prognostic markers.

## DNA methylation

DNA methylation is a chemical modification characterized by the covalent transfer of a methyl group at the C5 position of a cytosine base, producing 5-methylcytosine (Skvortsova et al., 2019). This modification occurs mainly at clusters of cytosine-guanine dinucleotides (CpG), named CpG islands (CGIs); is catalyzed by an enzymatic family called DNA methyltransferases (DNMTs); and plays an important role in gene expression regulation, genomic imprinting, X chromosome inactivation, retroelement silencing, and genome stability (Nishiyama and Nakanishi, 2021).

**Figure 2 -:**
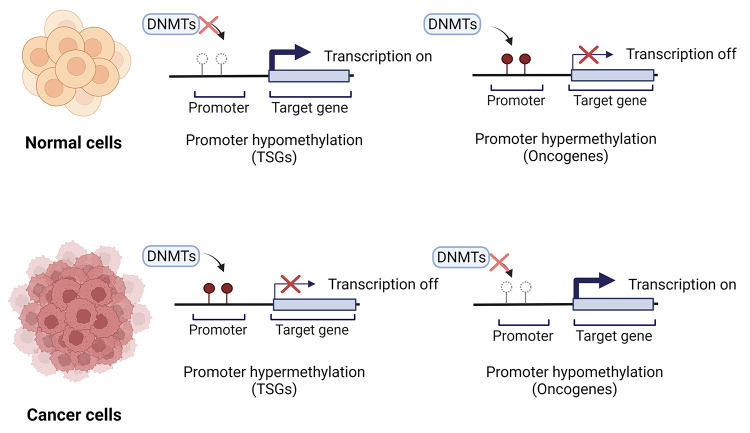
DNA methylation pattern in different cell types. In normal cells, the promoter regions of tumor suppressor genes (TSGs), as cell cycle regulators, are hypomethylated; meanwhile, in oncogenes (as transcription factors), this same region is frequently hypermethylated by DNA methyltransferases (DNMTs). On the other hand, an opposite pattern is described in cancer cells, with TSGs being hypermethylated, while oncogenes present a hypomethylated promoter region. Adapted from “Epigenetic deregulation in cancer”, by BioRender.com (2022). Retrieved from https://app.biorender.com/biorender-templates.

In normal cells, methylation is necessary for maintaining cell growth and metabolism, whereas an abnormal DNA methylation pattern can lead to diseases, such as cancers (Zhou et al., 2021). In cancer, hypermethylation of CGIs is common and frequently observed in transcriptional regulatory regions, such as promoters of tumor suppressor genes (TSGs), and is associated with the silencing of genes controlling cell growth and related pathways (Figure 2) (Skvortsova et al., 2019). Nevertheless, a DNA hypermethylation pattern is observed in a tissue-specific manner (Nishiyama and Nakanishi, 2021).

Although CpG dinucleotides are largely underrepresented in mammalian genomes, dogs have the largest number of CGIs and the highest CGI density among several mammalian genomes studied, such as humans, cows, and rats (Han and Zhao, 2009). Approximately 30,000 CGIs have been identified in the human genome (Jeziorska et al., 2017), whereas 58,327 CGIs have been mapped in the dog genome (Han and Zhao, 2009), with 2.2% of them annotated in the promoter and exon regions (Jeong et al., 2019), thus suggesting that DNA methylation must have some relevance on gene expression regulation in the dog genome.

To determine the influence of methylation in canine mammary carcinogenesis, Nam et al. (2020) analyzed only the CMT-associated genome-wide methylation signature using methyl CpG binding domain sequencing, observing that differentially methylated regions (DMRs) were distributed similarly on CGIs and tended to be enriched in gene regions. The authors described a hypermethylated pattern in tumoral tissues compared with their normal counterparts. In addition, hypermethylated regions were observed in TSGs, whereas oncogenes are frequently hypomethylated, especially in intronic regions, a result that is correlated with gene expression and with human breast cancer (Nam *et al.*, 2020).

A similar approach using the identification of DMRs to map genes subject to methylation modulation was applied by Schabort et al. (2020), which resulted in a mapping of 16,061 differentially methylated genes. Among them, two genes, *ANK2* and *EPAS1*, were observed to have a high level of hypermethylation in tumoral tissues compared with normal samples. In addition, a reverse correlation between the hypermethylation status and gene expression was described, suggesting that the expression levels of those genes are affected by their methylation status. Although both genes could be used as biomarkers in mammary tissues, in plasma samples this same profile was observed only for *ANK2*, similar to the described for human breast cancer, highlighting its potential as a diagnostic marker in liquid biopsy.

Although the classification in molecular subtypes is not frequently observed in CMTs, in a work focusing on the triple-negative (TN) subtype of mammary cancer, Beetch et al. (2020) analyzed samples of different tumor stages as well as healthy donors, described the hypermethylation pattern of cancer-related genes, and found that those involved in transcriptional regulation, apoptosis, signal transduction, and cell migration are associated with gene expression and progression of the disease in dogs, suggesting that the methylation pattern has the potential to be utilized in clinics to distinguish progression stages in TN cancers.

In a broader analysis, Biondi *et al.* (2021), who analyzed the global genome methylation pattern of canine mammary tumors by immunohistochemistry, described a lower hypermethylation pattern for malignant samples, which is associated with tumor recurrence. In the same way, in mammary tissues as well as in plasma, Lee et al. (2019) observed a low methylation level in LINE-1, a retrotransposon, in benign and tumor samples when compared with healthy samples. Although these results might seem contradictory with the previous ones, they are in accordance with the global hypomethylated and regional hypermethylated patterns in tumors during their initiation and progression.

Meanwhile, studies on mammary cancer evaluating the methylation pattern of single genes did not find any evidence of methylation affecting gene expression regulation, as described for *BRCA1* (Qiu and Lind, 2016; Ferreira et al., 2019), *ESR1* (Brandão et al., 2018) and *P15/CDKN2B* (Faro et al., 2018). These results differ slightly from those observed in human counterparts. For *CDKN2B*, there are still contradictory results, ranging from no correlation between promoter hypermethylation and breast cancer risk (Qi and Xiong, 2018) to an early and frequent event of breast carcinogenesis (Jung et al., 2013). The hypermethylation of the promoter regions of *BRCA1* (Jung *et al.*, 2013; Harahap et al., 2018; Vu et al., 2018) and *ESR1* (Sun et al., 2017; Kirn et al., 2018), which are considered biomarkers, is associated with breast carcinogenesis. On the other hand, Ren et al. (2018), who analyzed the methylation pattern of *DAPK1* and *MGMT* genes in clinical samples, observed an increase in methylation status according to tumor aggressiveness, suggesting their use as prognostic and diagnostic markers, as observed in humans (An et al., 2017; Yadav et al., 2018; Ghalkhani et al., 2021; Yari et al., 2022).

The alterations observed in cancer methylation patterns may be caused by DNMT expression dysregulation; thus, several anticancer drugs targeting DNMTs, such as the DNA methyltransferase inhibitor 5-azacitydine (5-AzaC), are approved for clinical use to treat tumors, such as acute myelodysplastic leukemia (Hillyar et al., 2020). In humans, 5-AzaC has been shown to inhibit the invasiveness and growth of several human breast cancer cell lines, and its potential as a therapeutic agent in canine mammary cancer was demonstrated by Harman et al. (2016), suggesting that more studies concerning the use of 5-AzaC in canine mammary cancer should be developed.

## Histone modifications

One of the features observed in eukaryotic cells is that, in the nucleus, DNA is packaged in the form of chromatin, a macromolecular complex of DNA and histone proteins (Dawson and Kouzarides, 2012). The chromatin has a basic unit called a nucleosome, which is formed by 147 base pairs of DNA wrapped around a histone octamer (comprising two of each histone: H2A, H2B, H3, and H4). The protruding N-terminal amino acid tails of core histones (especially H3 and H4) are subject to posttranslational modifications (PTMs), including methylation, acetylation, phosphorylation, ubiquitination, sumoylation, and ADP ribosylation, which can regulate the chromatin structure and remodel it, resulting in two structural and transcriptional states according to the noncovalent interactions within and between nucleosomes: an active and a repressive state (Dawson and Kouzarides, 2012; Zhang et al., 2015; Ilango et al., 2020; Boyson et al., 2021).

**Figure 3 -:**
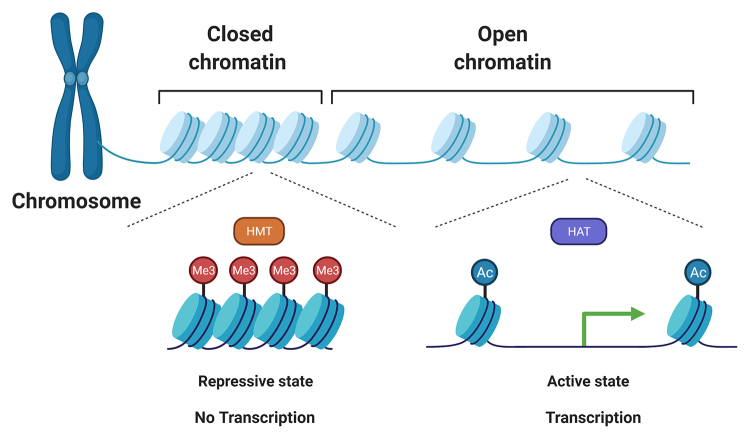
Histone modifications and their impact on chromatin remodeling. Histone methylation is catalyzed by histone methyltransferases (HMTs), resulting in chromatin packaging (heterochromatin) and a repressive state (no transcription). Conversely, histone acetylation by histone acetyltransferases (HATs) results in open chromatin (euchromatin) associated with an active transcriptional pattern. Adapted from “Epigenetics and gene expression”, by BioRender.com (2022). Retrieved from https://app.biorender.com/biorender-templates.

While the repressive states are formed by supercoiled structures enriched for DNA and histone methylation marks (such as H3 trimethylation of lysine 27 and lysine 9), resulting in closed chromatin (heterochromatin), active states are accessible to transcription factors and are enriched for histone marks, such as high levels of lysine acetylation on the H3 and H4 tails, which forms open chromatin (euchromatin) frequently associated with an active transcriptional pattern (Figure 3) (Biswas and Rao, 2018; Nebbioso et al., 2018). These patterns of histone marks are established through a dynamic interplay between protein machineries that recognize, add, and remove these PTMs, named histone readers, writers, and erasers, respectively (Gillette and Hill, 2015; Zhang et al., 2015; Uckelmann and Davidovich, 2021).

Histone writers, such as histone methyltransferases (HMTs) and histone acetyltransferases (HATs), are a group of enzymes capable of transferring chemical compounds, such as methyl and acetyl groups, to the N-terminal tail of histones. These covalent modifications are thus recognized and interpreted by histone readers, such as BET proteins, which are capable of identifying and binding due to the presence of specialized domains, inducing chromatin structural changes, recruiting other chromatin modifiers, or providing scaffold proteins for different nuclear processes, such as transcription, replication, or repair. On the other hand, those PTMs may be removed by enzymes known as erasers, such as histone demethylases (HDMs) and histone deacetylases (HDACs) (Biswas and Rao, 2018; López et al., 2022).

These modifications play an important role in human breast cancer, providing specific chromatin signatures for each molecular subtype (Xi et al., 2018), and could be considered prognostic (Elsheikh et al., 2009; Fath et al., 2022) and therapeutic (Sharda et al., 2020; Wang et al., 2020) markers. In canine mammary cancer, as stated by Liu et al. (2014), 35 downregulated chromatin-modifier genes, mostly related to histone active and repressive modifiers (i.e., associated with methylation, demethylation, acetylation, deacetylation, ubiquitination, and deubiquitination modifications), have been described in complex carcinomas with myoepithelial cell proliferation, suggesting that this tumor type originates mainly from epigenomic rather than genomic alterations.

BET proteins are a family of epigenetic readers characterized by two tandem N-terminal BRD regions followed by an extraterminal domain, which bind acetylated histones, modulate chromatin architecture and recruit transcriptional regulators (Sahni and Keri, 2018; Hill et al., 2021; Neganova et al., 2022). The use of BET protein inhibitors, already described as potential therapeutic agents in human breast cancer (Andrikopoulou et al., 2020; Khandekar and Tiriveedhi, 2020), is also a promising therapy in canine mammary cancer, as they can downregulate several genes related to self-renewal pathways, such as WNT, NOTCH, Hedgehog, and PI3K/AKT/mTOR (Xavier et al., 2019).

Meanwhile, analysis of histone modifications considering different tumor grades obtained contrasting results. Although Liu et al. (2014) did not correlate their findings with tumor grade, Vinothini et al. (2009) described significantly increased expression in histone deacetylase 1, a chromatin-modifier eraser protein, in tumors in more advanced states (grade III) than in those in more initial states (grades I and II). Similar results were described for EZH2, the catalytic subunit of the epigenetic regulator Polycomb repressive complex 2, a protein complex involved in gene silencing through the trimethylation of histone 3 lysine residue 27, where EZH2 expression increases with the malignancy grade (Choi et al., 2016), suggesting that this alteration may also influence the prognosis of the patient. Although not related to tumor grade, EZH2 upregulation in human breast cancer is associated with a poor prognosis and is a potential target for therapeutic options (Xie et al., 2022; Zhang et al., 2022).

## Noncoding RNAs

Noncoding RNAs (ncRNAs) are regulatory players of gene expression classified according to their size and function, which includes small interfering RNAs, microRNAs (miRNAs), and long ncRNAs (lncRNAs), playing important roles in gene expression regulation at several levels: transcription, mRNA degradation, splicing, and translation (Inbar-Feigenberg et al., 2013).

miRNAs are short single-stranded ncRNAs approximately 17-24 nucleotides in length that posttranscriptionally regulate gene expression and are involved in the regulation of several cellular pathways, such as differentiation, proliferation, and apoptosis (Heishima et al., 2017; Loh et al., 2019; Pasquini and Kunej, 2019). Dysregulation of a single miRNA or a small subset of miRNAs can therefore have a significant impact on cellular outcomes and sometimes induce the development of several disease processes, such as cancer (Loh *et al.*, 2019). Considering their dysregulation during tumorigenesis, miRNAs can be subdivided into two main classes: oncogenic miRNAs (oncomiRs), usually upregulated and responsible for suppressing the expression of TSGs, and tumor suppressor miRNAs, a class that inhibits the expression of oncogenes and is usually downregulated in cancer (Loh *et al.*, 2019). However, some miRNAs may play a dual role depending on the tumor site (Kolenda et al., 2020).

One feature of cancer cells is that they can release their miRNAs into the bloodstream. Such “circulating miRNAs” are stabilized by either their binding proteins or extracellular vesicles, such as exosomes and microvesicles, which makes them resistant to RNase degradation. As the levels of circulating miRNAs accurately reflect the number of tumor cells, response to treatment, clinical stages, and tumor grades, circulating miRNAs have a great potential to be used as diagnostic and prognostic biomarkers in neoplastic diseases (Heishima et al., 2017).

Due to its relevance in cellular pathways, miRNA expression is the most studied epigenetic alteration in canine mammary cancer and is considered a promising biomarker (Table 1). However, in this review, we will focus on the most studied miRNAs and their relevance to canine mammary tumorigenesis.

**Table 1 -:** miRNA expression pattern in clinical samples of canine mammary cancer and their possible application on veterinary routine. CL= cell line; P= plasma; S= serum; T= mammary tissue.

miRNA	Sample type	Expression	Clinical application	References
miR-18a	S	Up-regulated	Prognostic	Fish et al. (2020)
miR-19b	S	Up-regulated	Diagnostic	Fish et al. (2020)
miR-21	T/S	Up-regulated	Diagnostic and prognostic	Boggs et al. (2008); von Deetzen et al. (2014); Bulkowska et al. (2017); Jain et al. (2021); Ramadan et al. (2021)
miR-29b	T/S	Down-regulated	Diagnostic and prognostic	Bulkowska et al. (2017); Jain et al. (2021)
	T/S	Up-regulated	Diagnostic	Boggs et al. (2008); Fish et al. (2020)
miR-96	T/S	Up-regulated	Diagnostic	Jeong et al. (2019)
miR-101	T/S	Down-regulated	Diagnostic	von Deetzen et al. (2014); Bulkowska et al. (2017)
miR-125a	T/S	Down-regulated	Prognostic	von Deetzen et al. (2014); Bulkowska et al. (2017)
	S	Up-regulated	Diagnostic	Fish et al. (2020)
miR-126	P	Up-regulated	Diagnostic	Heishima et al. (2017)
miR-143	T/S	Down-regulated	Prognostic	von Deetzen et al. (2014); Bulkowska et al. (2017)
miR-145	T/S	Down-regulated	Prognostic	von Deetzen et al. (2014); Bulkowska et al. (2017)
miR-149	T/S	Down-regulated	Diagnostic	Jeong et al. (2019)
miR-194	T	Up-regulated	Diagnostic	von Deetzen et al. (2014)
miR-195	T/CL	Down-regulated	Diagnostic	Zhang *et al.* (2021)
miR-210	T/S	Down-regulated	Diagnostic	von Deetzen et al. (2014); Bulkowska et al. (2017)
miR-214	P	Up-regulated	Diagnostic	Heishima et al. (2017)
miR-497	T/CL	Down-regulated	Diagnostic	Zhang *et al.* (2021)
miR-8832	T/S	Up-regulated	Diagnostic	Jeong et al. (2019)

One of the most studied miRNAs is miR-21, which is described as an oncomiR in humans and is involved in cell migration, invasion, metastasis, and apoptosis (Nurzadeh et al., 2021). Although its characterization in a cell line derived from a grade II mammary tumor suggests a low expression of miR-21 (Raposo et al., 2017), the same pathogenic role described for humans was observed by Boggs et al. (2008) and von Deetzen et al. (2014), who reinforced the oncogenic role of this miRNA in mammary cancer. Therefore, different studies have analyzed miR-21 expression in the serum samples of patients with mammary cancer, comparing the results with either or both patients who are healthy and with benign tumors. Overall, overexpression of miR-21 is observed in patients with cancer, supporting its use as a diagnostic and prognostic marker using minimally invasive methods (Hannafon et al., 2016; Jain et al., 2021; Ramadan et al., 2021).

Another canine mammary cancer studied is miR-18a, which is upregulated in human breast cancer, although it can be downregulated in other tumor types, such as gastric cancer (Kolenda et al., 2020). In canines, this miRNA is an oncomiR that is upregulated in exosomes isolated from mammary cell lines (Fish et al., 2018) and in body fluids of tumoral patients, where it is also associated with lymph node invasion (Fish *et al.*, 2020), suggesting its use as a diagnostic and prognostic marker in liquid biopsy. This miRNA may be involved in other levels of epigenetic regulation, as several of their gene targets are related to chromatin remodeling processes, such as methylation, acetylation, and ubiquitination of histones (Fish *et al.*, 2018).

Although the majority of studies are in agreement with the function of the miRNAs in canine mammary cancer, controversial results exist in some cases; for example, miR-29b is downregulated in the FR37-CMT cell line (Raposo et al., 2017) and in tumoral tissues of canine patients (Jain et al., 2021) but upregulated in the SNP cell line (Osaki et al., 2016), tumoral tissues (Boggs et al., 2008), and serum (Fish et al., 2020) of patients.

lncRNAs are molecules with at least 200 nucleotides transcribed mainly by RNA polymerase II that do not encode proteins and can influence gene expression at the transcriptional and posttranscriptional levels, influencing several cellular processes, such as proliferation, differentiation and apoptosis (Naz et al., 2021; Smolarz et al., 2021). Despite a few studies in the area, lncRNAs have great potential as predictive markers of canine mammary cancer. Xu et al. (2021) reported that the overexpression of lnc-42060 in tumoral tissues and mammary cancer cell lines is associated with cell proliferation, migration, and increased tamoxifen resistance. A similar result was described by Lu et al. (2022), who analyzed cell lines and tissues of patients with mammary cancer and described the influence of two lncRNAs (the downregulated expression of lnc-40589 and overexpressed lnc-34977) on cell proliferation, migration, and invasion in mammary cancer, suggesting a key role during carcinogenesis.

## Conclusion

Although canine studies are still scarce compared with those of human counterparts, the studies approaching the epigenetic aspects of canine mammary cancer reinforced the importance of canine mammary carcinogenesis. Several described epigenetic alterations, especially those involving ncRNAs, have potential as predictive, diagnostic, and prognostic markers for canine mammary cancer, including its use in liquid biopsy. These biomarkers may be further used in clinical routines and likely help veterinary doctors proceed on a faster and more accurate diagnosis and choose the best treatment option for their patients. Considering the above, larger and more detailed studies in the epigenetic field are urgently needed to better understand the influence of this area on canine mammary tumors.
